# How do fertility facilities in Japan perceive disclosing institutional success rates for IVF? A nationwide survey of registered assisted reproductive technology facilities

**DOI:** 10.1002/rmb2.12653

**Published:** 2025-06-12

**Authors:** Seung Chik Jwa, Eri Maeda, Osamu Ishihara, Akira Tsujimura, Yukihiro Terada, Yutaka Osuga

**Affiliations:** ^1^ Department of Obstetrics and Gynecology Jichi Medical University Shimotsuke Japan; ^2^ Department of Public Health, Faculty of Medicine Hokkaido University Sapporo Japan; ^3^ Nutrition Clinic Kagawa Nutrition University Saitama Japan; ^4^ Department of Urology Juntendo University Urayasu Hospital Urayasu Japan; ^5^ Department of Obstetrics and Gynecology Akita University Graduate School of Medicine Akita Japan; ^6^ Department of Obstetrics and Gynecology, Graduate School of Medicine The University of Tokyo Tokyo Japan

**Keywords:** assisted reproductive technology, embryo transfer, in vitro fertilization, pregnancy rate

## Abstract

To evaluate the current perspectives on reporting success rates for assisted reproductive technology, the authors conducted a nationwide survey. Of the 327 facilities that responded (response rate: 53.5%), over half recognized potential benefits of reporting in vitro fertilization (IVF) success rates, such as aiding patients in selecting a clinic (68.5%) and enhancing the quality and efficiency of information provided to patients (62.1%). However, concerns regarding potential negative impacts, including patient selection bias, were also highlighted, albeit to a lesser extent (32.7%–52.3%). These findings underscore the need for further discussions to establish an unbiased reporting framework and improve patient education on assisted reproductive technology (ART) success rates.

## Graphical abstract

This survey provides the first comprehensive evaluation of Japanese ART facilities' attitudes toward IVF success rate disclosure. The findings indicate a predominantly positive perception of such reporting. Based on these insights, further discussions are warranted to establish unbiased reporting systems and robust patient education initiatives to facilitate accurate interpretation of disclosed information.

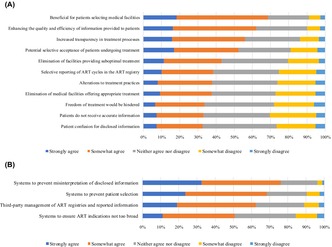

## 1 INTRODUCTION

In Japan, approximately 543 630 cycles of in vitro fertilization (IVF) and other assisted reproductive technologies (ART) are performed annually,^1^ making the country the second‐largest performer of ART cycles globally following China. When adjusted for population size, Japan exhibits one of the highest utilization rates of ART cycles worldwide.^2^ Since the incorporation of ART into the national health insurance system in April 2022, the associated out‐of‐pocket expenses have decreased substantially, leading to a notable increase in the number of treatment cycles performed during 2022.

Despite the extensive availability of ART services in Japan, the information accessible to patients for selecting appropriate ART facilities remains limited. Currently, the only standardized and publicly accessible data on ART facilities are provided through a government‐run Web site designed for the specific infertility treatment support program linked to the former governmental subsidy scheme.^3^ This platform provides limited basic treatment details, such as the types of infertility treatments offered, staffing information, and, optionally, data on the number of oocyte pick‐up procedures, embryo transfers (ET), and live births. However, critical outcome indicators—such as pregnancy or live birth rates per ET stratified by patient age—are notably absent.

The publication of facility‐specific treatment outcomes holds promise as a valuable resource for patients. However, interpreting these metrics requires careful consideration of various confounding factors, including patient characteristics, such as age, which vary across facilities. To date, no comprehensive study has been conducted to evaluate the attitudes of ART facilities toward the disclosure of treatment information, including facility‐specific success rates.

To address this gap, the present study undertook a nationwide survey targeting ART facilities registered with the Japan Society of Obstetrics and Gynecology (JSOG). The objectives were to assess current practices regarding the disclosure of treatment information and to explore the perspectives of facilities on reporting institutional IVF success rates.

## 2 MATERIALS AND METHODS

This nationwide web‐based survey targeted all ART facilities in Japan. A letter containing a unique ID and password for online questionnaire access was distributed to all 611 ART facilities registered with the JSOG in February 2023. Of these, 327 facilities responded between February 20 and April 13, 2023, yielding a response rate of 53.5%. The detailed study protocol and actual questionnaire used for the study are available in Supporting Information.

The survey collected detailed information in three domains: (1) characteristics of ART facilities, including geographic location, facility type (clinic or hospital), presence of a delivery unit, and the total annual number of ART cycles; (2) the current status of treatment information disclosure, encompassing reports to the government's specific infertility treatment support program as well as dissemination through facility Web sites or printed materials; and (3) the attitudes of ART facilities toward potential changes arising from the disclosure of treatment information and success rates. Responses regarding potential changes were assessed using a 5‐point Likert scale (strongly agree, somewhat agree, neither agree nor disagree, somewhat disagree, strongly disagree) for the following considerations: (1) facilitation of patient clinic selection; (2) enhancement of the quality and efficiency of patient information; (3) increased treatment transparency; (4) potential selective acceptance of patients undergoing treatment; (5) elimination of facilities providing suboptimal treatment; (6) potential selective registration of ART cycles; (7) modifications in treatment practices; (8) possible elimination of facilities providing appropriate treatment; (9) restrictions on treatment freedom; (10) patients do not receive accurate information; and (11) patient confusion for disclosed information, and others.

Descriptive analyses were conducted to evaluate the current practices and perceptions of ART facilities regarding the reporting of treatment information, including success rates. Furthermore, we classified ordinance‐designated cities, core cities, and special wards as urban areas, while other regions were considered rural areas. Using this classification, we analyzed whether there were differences in the attitudes of ART facilities toward potential changes arising from the disclosure of treatment information and success rates between urban and rural areas using the chi‐squared test or Fisher's exact test as appropriate. All statistical analyses were performed using Stata/MP, version 17.0 (StataCorp LLC, College Station, TX, USA).

## 3 RESULTS

The characteristics of the facilities included in this study are summarized in Table S1. Among the responding institutions, 116 (35.5%) were hospitals, while 211 (64.5%) were clinics. A total of 226 facilities (40.0%) were located within special wards, ordinance‐designated cities, or core cities. The median annual number of ART treatment cycles was 546, with an interquartile range (IQR) of 247‐1226.

Table 1 summarizes the disclosure status of optional items related to the government‐specific infertility treatment support program. The most frequently disclosed parameter was the number of frozen–thawed embryo transfers (FET), reported by 72.5% of facilities. This was followed by the number of pregnancies achieved through FET (68.2%), with similarly high disclosure rates for the number of oocyte retrieval cycles (65.4%) and pregnancies achieved in fresh ET cycles (60.6%). In contrast, disclosure rates for treatment data categorized by fertilization method, such as IVF and intracytoplasmic sperm injection (ICSI), were markedly lower, ranging from 26.3% to 35.5%. Additionally, disclosure rates for live births (45.0%–45.9%) and live birth rates per ET (45.3%–48.6%) were lower than those for treatment cycles and pregnancy outcomes in fresh and frozen cycles.

**TABLE 1:** Current disclosure status of optional items for the governmental specific infertility treatment support program (*n* = 327).

	*n* (%)
Fresh cycles	
Total number of oocyte pick‐up	214 (65.4)
IVF	116 (35.5)
ICSI	113 (34.6)
Split‐ICSI	86 (26.3)
None‐reporting	106 (32.4)
Total number of embryo transfers	219 (67.0)
IVF	99 (30.3)
ICSI	96 (29.4)
Split‐ICSI	73 (22.3)
None‐reporting	105 (32.1)
Total number of pregnancy	198 (60.6)
IVF	96 (29.4)
ICSI	94 (28.8)
Split‐ICSI	72 (22.0)
None‐reporting	105 (32.1)
Total number of live birth	147 (45.0)
IVF	81 (24.8)
ICSI	79 (24.2)
Split‐ICSI	65 (19.9)
None‐reporting	176 (53.8)
Live birth rate per embryo transfers	148 (45.3)
IVF	87 (26.6)
ICSI	85 (26.0)
Split‐ICSI	67 (20.5)
None‐reporting	174 (53.2)
Frozen cycles	
Total number of embryo transfers	237 (72.5)
Total number of pregnancy	223 (68.2)
Total number of live birth	150 (45.9)
Live birth rate per embryo transfers	159 (48.6)

The current status of public reporting of treatment outcomes at individual facilities is detailed in Table S2. Of the surveyed facilities, 183 (56.0%) indicated that treatment outcomes were disclosed either on their Web sites or in printed materials at their locations. Among these facilities, the most commonly disclosed metric was the pregnancy rate for FET (92.9%), followed by the pregnancy rate for fresh ET (67.8%). However, live birth rates per ET were disclosed less frequently, with rates of 35.5% for FET and 25.7% for fresh ET. Notably, 31 facilities (16.9%) reported, including chemical pregnancies in their success rate calculations (Table S2).

**FIGURE 1:**
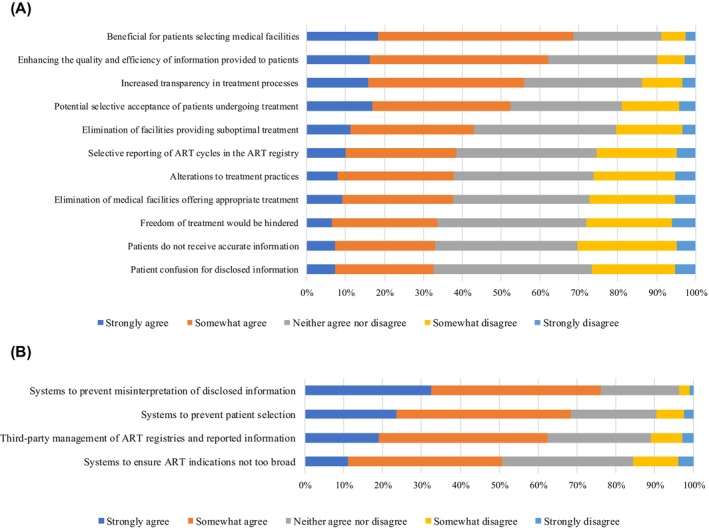
(A) Attitudes of assisted reproductive technology (ART) facilities toward potential changes resulting from disclosing treatment success rates. (B) Need for appropriate disclosure of treatment information and success rates (*n* = 327). ART, assisted reproductive technology.

The potential impacts of disclosing treatment success rates are illustrated in Figure 1A. A majority of facilities (68%) agreed (combining “strongly agree” and “somewhat agree”) that such disclosures are beneficial for patients selecting medical facilities. Other perceived benefits included improvements in the quality and efficiency of information provided (62%) and increased transparency in treatment processes (56%). Conversely, fewer facilities agreed with potential negative impacts, such as the risk of patient selection bias (52%), the elimination of medical facilities perceived as offering suboptimal treatment (43%), selective reporting of ART cycles in the registry (39%), and alterations to treatment protocols (38%). These responses did not differ between urban and rural areas based on the facilities' location (Table S3). Lastly, preferences for the source of success rate reporting in ART were assessed. Most facilities (86.2%; 282 facilities) indicated a preference for using data from the ART registry over individual facility reports (33.3%, 109 facilities) (Table S4).

## 4 DISCUSSION

A key finding of this study is that 68.5% of ART facilities recognized the potential value of disclosing IVF success rates, indicating a shared understanding of the growing demand among patients for transparent and reliable information to inform clinic selection. Moreover, 62.1% of respondents acknowledged that such disclosure could enhance the quality and accessibility of information for patients, underscoring the facilities' recognition of the critical role of informed decision‐making in patient care.

However, the survey also identified notable concerns. Specifically, 52.3% of facilities expressed apprehension that disclosing success rates might incentivize selective patient acceptance, in which clinics could avoid high‐risk cases to maintain favorable statistics. Similarly, 39% of respondents raised concerns about the selective reporting of cycles in the ART registry. These risks have been reported in other countries as well.^4^ For example, in the United States, the Fertility Clinic Success Rate and Certification Act of 1992 mandated ART success rate reporting, but the retrospective nature of data collection introduced vulnerabilities to data omissions and manipulation. Williams et al.^4^ proposed mandatory prospective data collection, such as that required by the Society for Assisted Reproductive Technology, which mandates the reporting of cycle initiation data within 4 days of the procedure's commencement. This prospective approach enhances accountability by safeguarding the denominator in success rate calculations, thereby reducing the likelihood of selective exclusion of poor‐prognosis cases.

The survey further revealed that the majority of facilities (86.2%) preferred the Japanese ART registry as a data source for calculating success rates over individual facility reporting (33.3%), reflecting a perception that registry data offers greater reliability and resistance to manipulation, providing a more accurate depiction of national trends and outcomes. Nevertheless, a significant proportion of respondents (41.0%) identified the absence of on‐site audits and quality control in the Japanese ART registry as a critical limitation. To ensure the registry's utility as a reliable data source, the integration of robust quality control measures and prospective data collection systems is essential.

One of the most frequently cited requirements for effective disclosure of treatment success rates was the establishment of a “system to prevent misinterpretation of disclosed information” (76.1%, 249 facilities, Figure 1B). This highlights the necessity of contextualizing reported outcomes. For example, the Human Fertilization and Embryology Authority in the UK provides explanatory guidelines for interpreting results via its Web site, an approach that could serve as a model for Japan.^5^ Such educational initiatives should accompany data disclosure to ensure accurate comprehension by patients.

The primary limitation of this study is that it captures only the subjective opinions of ART facilities, without incorporating objective clinical data or infertility patient perspectives. Additionally, this survey does not assess whether patients actively select ART facilities based on disclosed success rates, making it unclear whether public reporting directly influences patient decision‐making or leads to improved clinical outcomes. However, as part of our ongoing research funded by the Ministry of Health, Labour and Welfare, we are currently conducting a separate survey targeting infertility patients to evaluate their perspectives on success rate disclosure. Integrating these patient insights and clinical data in future studies will be essential for a more comprehensive understanding of the impact of disclosure on both patient choices and facility practices.

In conclusion, this survey provides the first comprehensive evaluation of Japanese ART facilities' attitudes toward IVF success rate disclosure. The findings indicate a predominantly positive perception of such reporting, tempered by concerns about potential misuse. While concrete solutions remain elusive, our results serve as a valuable starting point for future policy discussions. Based on these insights, further discussions are warranted to establish unbiased reporting systems and robust patient education initiatives to facilitate accurate interpretation of disclosed information.

## CONFLICT OF INTEREST STATEMENT

The authors declare no conflict of interest. “Seung Chik Jwa” and “Akira Tsujimura” are an Editorial Board member of *Reproductive Medicine and Biology* and a coauthor of this article. To minimize bias, they are excluded from all editorial decision‐making related to the acceptance of this article for publication.

## ETHICS STATEMENT

Human Rights Statement and Informed Consent: All procedures were performed in accordance with ethical standards of the institutional committees on human experimentation (institutional and national) and the Helsinki Declaration of 1964 and its later amendments.

Animal Studies: This article does not contain any studies with animal subjects performed by any of the authors.

Approval by Ethics Committee: The survey targeted medical facilities and did not fall under “Ethical Guidelines for Life Science and Medical Research Involving Human Subjects.” Therefore, ethics approval was exempt for this study.

## Supporting information


Data S1:
