# Supplementary material for: How do fertility facilities in Japan perceive disclosing institutional success rates for IVF? A nationwide survey of registered assisted reproductive technology facilities
Source: Reprod Med Biol. 2025 Jun 12;24(1):e12653. doi: 10.1002/rmb2.12653 (PMC12159763; doi:10.1002/rmb2.12653)
Supplement: Supplementary file 1 — Data S1: [file RMB2-24-e12653-s001.docx]

**Supplemental materials**

1. **Supplemental Table 1. Characteristics of the participating ART facilities (n = 327).**
2. **Supplemental Table 2. Current reporting status and definition of pregnancy for treatment information reporting to the public by individual facilities (n = 327).**
3. **Supplemental Table 3. Attitudes of ART facilities toward potential changes due to the disclosure of treatment success rates, stratified by facility location (n = 327).**
4. **Supplemental Table 4. Preferred information sources and institutional bodies for disclosing treatment information and outcomes by individual facilities (n = 327).**
5. **Study protocol**
6. **Survey questionnaire used for the study**

| Supplemental Table 1. Characteristics of the participating ART facilities (n = 327). | |
| --- | --- |
|  | n (%) |
| **Types of facility** |  |
| Hospital | 116 (35.5) |
| Clinic | 211 (64.5) |
| **Facility treating delivery** | 142 (43.3) |
| **Proportions of infertility patients** |  |
| more than 70-80% | 160 (48.9) |
| around 50% | 65 (19.9) |
| 10-20% | 102 (31.2) |
| **Location of facility** |  |
| Special ward | 49 (15.0) |
| Ordinance-designated city | 87 (26.6) |
| Core city | 90 (27.5) |
| Other city | 92 (28.1) |
| Town or village | 9 (2.8) |
| **Governing body** |  |
| National | 31 (9.5) |
| Public | 21 (6.4) |
| Social insurance organization | 6 (1.8) |
| Medical corporation | 193 (59.0) |
| Individual | 46 (14.1) |
| Others | 30 (9.2) |
| **Total ART treatment cycles^a^** |  |
| <100 | 40 (12.2) |
| 100-300 | 60 (18.4) |
| 300-500 | 54 (16.5) |
| 500-1000 | 76 (23.2) |
| more than 1000 | 97 (29.6) |
| ^a^ Total treatment cycles of oocyte pick-up and frozen-thawed embryo transfer cycles. | |
| ART, assisted reproductive technology. |  |

| Supplemental Table 2. Current reporting status and definition of pregnancy for treatment information reporting to the public by individual facilities (n = 327). | |
| --- | --- |
|  | n (%) |
| None-reporting | 144 (44.0) |
| Reporting | 183 (56.0) |
| IUI^a^ |  |
| Pregnancy rate | 88 (48.1) |
| Live birth rate | 17 (9.3) |
| Fresh cycle^a^ |  |
| Fertilization rate | 29 (15.9) |
| Blastocyst development rate | 27 (14.8) |
| Pregnancy rate per embryo transfer | 124 (67.8) |
| Live birth rate per embryo transfer | 47 (25.7) |
| Frozen cycles^a^ |  |
| Rate of successful embryo transfer | 53 (29.0) |
| Pregnancy rate per embryo transfer | 170 (92.9) |
| Live birth rate per embryo transfer | 65 (35.5) |
| Definition of pregnancy for success rate^a^ |  |
| Biochemical pregnancy | 31 (16.9) |
| Confirmation of gestational sac | 172 (94.0) |
| Confirmation of fetal heart beat | 32 (17.5) |
| ^a^ Denominators represent the number of facilities reporting treatment information to the public (n = 183). | |

| Supplemental Table 3. Attitudes of ART facilities toward potential changes due to the disclosure of treatment success rates, stratified by facility location (n = 327). | | | | | | | | | | | | |
| --- | --- | --- | --- | --- | --- | --- | --- | --- | --- | --- | --- | --- |
|  | Urban area (n = 226)^a^ | | | | |  | Rural area (n = 101)^a^ | | | | | |
|  | Strongly agree | Somewhat agree | Neither agree nor disagree | Somewhat disagree | Strongly disagree |  | Strongly agree | Somewhat agree | Neither agree nor disagree | Somewhat disagree | Strongly disagree | P-value^b^ |
| Beneficial for patients selecting medical facilities | 40 (17.7) | 118 (52.2) | 45 (19.9) | 16 (7.1) | 7 (3.1) |  | 20 (19.8) | 46 (45.5) | 29 (28.7) | 5 (5.0) | 1 (1.0) | 0.33 |
| Enhancing the quality and efficiency of information provided to patients | 35 (15.5) | 106 (46.9) | 62 (27.4) | 16 (7.1) | 7 (3.1) |  | 18 (17.8) | 44 (43.6) | 30 (29.7) | 7 (6.9) | 2 (2.0) | 0.94 |
| Increased transparency in treatment processes | 35 (15.5) | 98 (43.4) | 63 (27.9) | 22 (9.7) | 8 (3.5) |  | 17 (16.8) | 33 (32.7) | 36 (35.6) | 12 (11.9) | 3 (3.0) | 0.41 |
| Potential selective acceptance of patients undergoing treatment | 39 (17.3) | 79 (35.0) | 62 (27.4) | 38 (16.8) | 8 (3.5) |  | 16 (15.8) | 37 (36.6) | 32 (31.7) | 10 (9.9) | 6 (5.9) | 0.42 |
| Elimination of facilities providing suboptimal treatment | 28 (12.4) | 73 (32.3) | 79 (35.0) | 39 (17.3) | 7 (3.1) |  | 9 (8.9) | 31 (30.7) | 40 (39.6) | 17 (16.8) | 4 (4.0) | 0.85 |
| Selective reporting of ART cycles in the ART registry | 23 (10.2) | 64 (28.3) | 79 (35.0) | 51 (22.6) | 9 (4.0) |  | 10 (9.9) | 29 (28.7) | 39 (38.6) | 16 (15.8) | 7 (6.9) | 0.54 |
| Alterations to treatment practices | 18 (8.0) | 66 (29.2) | 80 (35.4) | 52 (23.0) | 10 (4.4) |  | 8 (7.9) | 32 (31.7) | 37 (36.6) | 17 (16.8) | 7 (6.9) | 0.69 |
| Elimination of medical facilities offering appropriate treatment | 22 (9.7) | 68 (30.1) | 73 (32.3) | 53 (23.5) | 10 (4.4) |  | 8 (7.9) | 25 (24.8) | 42 (41.6) | 19 (18.8) | 7 (6.9) | 0.38 |
| Freedom of treatment would be hindered | 14 (6.2) | 62 (27.4) | 85 (37.6) | 53 (23.5) | 12 (5.3) |  | 7 (6.9) | 27 (26.7) | 40 (39.6) | 19 (18.8) | 8 (7.9) | 0.81 |
| Patients do not receive accurate information | 16 (7.1) | 55 (24.3) | 84 (37.2) | 61 (27.0) | 10 (4.4) |  | 8 (7.9) | 29 (28.7) | 36 (35.6) | 22 (21.8) | 6 (5.9) | 0.77 |
| Patient confusion for disclosed information | 19 (8.4) | 52 (23.0) | 96 (42.5) | 49 (21.7) | 10 (4.4) |  | 5 (5.0) | 31 (30.7) | 37 (36.6) | 21 (20.8) | 7 (6.9) | 0.38 |
| ^a^ Urban area was defined if facilities were located in ordinance-designated cities, core cities, and special wards, while other regions were considered rural areas. | | | | | | | | | | | | |
| ^b^ Calculated using Chi-squared test or Fisher's exact test as appropriate. |  |  |  |  |  |  |  |  |  |  |  |  |

| Supplemental Table 4. Preferred information sources and institutional bodies for disclosing treatment information and outcomes by individual facilities (n = 327). | |
| --- | --- |
|  | n (%) |
| **Institutional body** |  |
| Government | 121 (37.1) |
| Academic Society | 232 (71.0) |
| Individual facility | 95 (29.1) |
| Others | 9 (2.8) |
| **Information source** |  |
| Japanese ART registry | 282 (86.2) |
| Receipt data for insured treatment | 57 (17.4) |
| Medical data within individual facility | 109 (33.3) |
| Others | 6 (1.8) |
| ART, assisted reproductive technology. | |

**5. Study protocol**

**Study Objectives:**

This study aims to:

1. Evaluate the current status of treatment outcome disclosure among assisted reproductive technology (ART) facilities in Japan.

2. Assess ART facilities’ perceptions and attitudes toward public reporting of treatment success rates.

3. Identify preferred organizations and data sources for managing outcome disclosure.

4. Explore potential challenges and necessary improvements to enhance transparency and standardization in reporting.

**Study Design:**

A cross-sectional survey was conducted to gather data from ART facilities regarding their disclosure practices and perspectives on public reporting.

**Study Population:**

• Target Population: ART facilities registered with the Japan Society of Obstetrics and Gynecology (JSOG)

•Number of Facilities Invited: 611

**Survey Period:**

• Data Collection Period: February 20 – April 13, 2023

**Data Collection Methods:**

• Survey Distribution:

• Web-based questionnaire

• Mailed invitation letters containing a unique ID and password for online access

**Survey Administration:**

• Conducted by an external research agency (Intage Inc.)

• Secure web-based response collection

**Ethical Considerations:**

• The study did not involve human subjects directly and was deemed exempt from ethical review under Japan’s guidelines for medical research involving human participants.

• This exemption was confirmed by the Saitama Medical University Ethics Committee before initiating the study.

1. **Survey questionnaire used for the study**
2. **貴施設について**
3. 貴施設の分類
   1. 病院
   2. 診療所
4. 分娩取り扱いの有無
5. 不妊治療の取り扱い状況
   1. 不妊治療の患者が大半である（全体の7～8割以上）
   2. 不妊治療の患者もいるが他の産婦人科の患者もいる（不妊治療の患者は全体の5割程度）
   3. 他の産婦人科の患者が大半である（不妊治療の患者は全体の1～2割）
6. 所在都道府県
7. 所在する市町村区分
   1. 特別区
   2. 政令指定都市
   3. 中核市
   4. その他の市
   5. 町村
8. 設立主体
   1. 国（国立大学法人含む）
   2. 公的医療機関（都道府県、市町村、地方独立行政法人、日赤、済生会等）
   3. 社会保険関係団体（健康保険組合及びその連合会、共済組合、国民健康保険組合）
   4. 医療法人
   5. 個人
   6. その他（公益法人、私立学校法人、社会福祉法人、医療生協、会社、その他法人）
9. 治療別年間実施周期数をご記入ください (2022年1月〜12月までに行った治療件数)
   1. タイミング指導
   2. 人工授精
   3. IVF-ET
   4. Split
   5. ICSI (射出精子)
   6. ICSI (TESE)
   7. FET
10. **貴施設における現時点での治療情報および成績の開示状況についてお尋ねします。**
    1. **不妊に悩む方への特定治療支援事業の実施医療機関における情報提供様式のうち、任意記載項目は以下のとおりです。貴施設が現在開示している項目を、すべてお選びください。**
11. 新鮮胚（卵）を用いた治療成績（総数　IVF　ICSI　Split別）
    - 1. 採卵周期数（総数　IVF　ICSI　Split別）
      2. 移植周期数（総数　IVF　ICSI　Split別）
      3. 妊娠数（総数　IVF　ICSI　Split別）
      4. 生産分娩数（総数　IVF　ICSI　Split別）
      5. 移植あたり生産率（総数　IVF　ICSI　Split別）
12. 凍結胚を用いた治療成績
    - 1. 移植周期数
      2. 妊娠数
      3. 生産分娩数
      4. 移植あたり生産率
13. IVF/ICSI/FET　年齢層別患者数
14. 精巣内精子採取術　年齢層別患者数
    1. **自施設のウェブサイトや紙媒体での成功率（妊娠率や生産率など）の公表状況についてお尋ねいたします。**
15. **貴施設はウェブサイトや紙媒体等を通じて、広く一般を対象に、治療の成功率を公開していますか？**
    1. いいえ（→本セクション終了、設問Ⅲへ）
    2. はい
16. **貴施設ではどの治療法について成功率を公開していますか？　当てはまるものをすべて選んでください。**
    1. 人工授精

妊娠率　/　生産率

- 1. 新鮮胚（卵）を用いた治療

受精率 / 胚盤胞到達率 / 妊娠率　/　生産率　/　その他

- 1. 凍結胚を用いた治療

移植成功率 / 妊娠率　/　生産率　/　その他

- 1. その他（　　　　　　　　　　　　　　　　）

1. **貴施設が成功率の開示に用いている「妊娠の定義」について、あてはまるものを　すべて選んでください。**
   1. 生化学的妊娠
   2. 胎嚢確認
   3. 胎児心拍確認
   4. その他（　　　　　　　　　）
2. **成功率（妊娠率・生産率）の分母についてお伺いします。貴施設が用いている分母をすべて選んでください。**
   1. 開始周期あたり
   2. 採卵周期あたり
   3. 移植周期あたり
   4. 初期胚移植周期あたり
   5. 胚盤胞移植周期あたり
   6. 治療患者あたり（累積）
   7. その他
3. **成功率に用いている「妊娠」の定義や、分母（治療周期や移植周期数等）の定義、いつ行われた治療であるかについて公表データに明記していますか。**

選択肢：

必ず記載している（開示データの100％）・

おおむね記載している（8割以上）・半数程度は記載している（4～6割）

あまり記載していない（2～3割）・全く記載していない（0～1割）

- 1. 妊娠の定義
  2. 分母の定義
  3. 治療実施時期（例：2020年の治療　等）

1. 中央社会保険医療協議会において、医療機関別の治療情報や成績の開示のあり方について検討するよう求められています。**医療機関別の治療情報や成績の開示について、貴施設がどのようにお考えになるかお伺いします。**
2. **医療機関別の治療情報や成績を開示すること全般についてご意見をお伺いします。貴施設のお考えに最も近いものを一つお選びください。**
   1. 施設の人員の配備の情報の開示について
      1. 全く賛成できない
      2. あまり賛成できない
      3. どちらともいえない
      4. ある程度賛成できる
      5. 大いに賛成できる
   2. 施設で導入している治療の実施状況の開示について　　　　　　　　　　　　　　 (先進医療導入の有無、実施件数等)
      1. 全く賛成できない
      2. あまり賛成できない
      3. どちらともいえない
      4. ある程度賛成できる
      5. 大いに賛成できる
   3. 治療件数の開示について
      1. 全く賛成できない
      2. あまり賛成できない
      3. どちらともいえない
      4. ある程度賛成できる
      5. 大いに賛成できる
   4. 多胎やOHSS等の合併症の件数や発生率の開示について
      1. 全く賛成できない
      2. あまり賛成できない
      3. どちらともいえない
      4. ある程度賛成できる
      5. 大いに賛成できる
   5. 治療の成功率（妊娠率や生産率）の開示について
      1. 全く賛成できない
      2. あまり賛成できない
      3. どちらともいえない
      4. ある程度賛成できる
      5. 大いに賛成できる
3. **次の各項目の開示は認められるとお考えになりますか？**

選択肢：　**認められない　・　条件付きで（※）認められる　・　認められる**

（※）「条件付きで」とは、たとえば

・数値だけでなく信頼区間を付けた範囲で示す

・症例数が少ない場合は、データの変動を考慮して、開示しない

・3段階などの区分で表示する　などの工夫を指します。

1. 新鮮胚（卵）を用いた治療成績（IVF　ICSI　Split別）
   - 1. 総治療周期数
     2. 採卵周期数
     3. 移植周期数
     4. 単一胚移植率
     5. 妊娠数
     6. 生産分娩数
     7. 総治療周期あたり妊娠率・生産率
     8. 採卵あたり妊娠率・生産率
     9. 移植あたり妊娠率・生産率
2. 凍結胚を用いた治療成績
   - 1. 移植周期数
     2. 単一胚移植率
     3. 妊娠数
     4. 生産分娩数
     5. 移植あたり妊娠率・生産率
3. 精巣内精子採取術　件数
4. **前問ⅱで「条件付きで認められる」とした方にお伺いします。どのような条件が必要だとお考えですか？当てはまる条件をすべてお選びください。**（それぞれについて回答）
   - 1. 数値だけでなく信頼区間を付けた範囲で示す
     2. 症例数が少ない場合は、データの変動を考慮して、開示しない
     3. 3段階などの区分で表示する
     4. 一定の年齢層の結果のみ開示する
     5. その他
5. **仮に、医療機関別の治療情報や成績を開示することになった場合、誰が、どのようなデータを使って、情報をまとめて、開示するのが望ましいでしょうか？　なお、現在は、各医療機関が自施設の情報を取りまとめていますが、用いるデータに関する制約は設けられていません。**
   1. 誰が、開示する情報を作成する（取りまとめる）のが望ましいですか？望ましいと思う機関をすべて選んでください。
      1. 国の機関（厚生労働省等）
      2. 学会（日本産科婦人科学会等）
      3. 各医療機関
      4. その他
   2. どのようなデータを使って情報をまとめるのが望ましいでしょうか。望ましいと思う情報源をすべて選んでください。
      1. 日本産科婦人科学会ARTオンライン登録データ
      2. レセプトデータ
      3. 各医療機関が持っている診療情報
      4. その他
6. **前問の理由について当てはまる理由をすべてお選びください。**
   1. 国や学会が情報を取りまとめるべきだとお考えになった理由（前問ⅳ（1）で①②を選んだ場合に表示）
      1. 公平性が保たれるから
      2. 不正を防げるから
      3. 自施設での公開の手間が大きいから
      4. 国または学会が、責任をもって行うべきだから
      5. 国または学会を、信用しているから
      6. その他
   2. 各医療機関が自施設について取りまとめるべきだとお考えになった理由（前問ⅳ（1）で③を選んだ場合に表示）
      1. 他機関（国や学会）による一方的な開示には不安があるから
      2. 患者に向けたメッセージや解釈を加えたいから
      3. 開示する項目を選びたいから
      4. 各施設で分担する方が効率的だから
      5. その他（　　　　　　　　　　　　　　　　　　　　　　　　）
   3. 日本産科婦人科学会ARTオンライン登録データまたはレセプトを情報源とすべきとお考えになった理由（前問ⅳ（2）で①②を選んだ場合に表示）
      1. 信頼できるから
      2. 不正を防げるから
      3. 効率的だから
      4. （日本産科婦人科学会ARTオンライン登録データには）妊娠・出産までのデータが入力されているから
      5. （レセプトデータで）保険診療分のART件数を明らかにすべきだから
      6. （レセプトデータで）ART以外の治療件数（人工授精等）も明らかにすべきだから
      7. その他（　　　　　　　　　　　　　　　　　　　　　　　　）
   4. 各施設の診療情報を情報源とすべきだとお考えになった理由（前問ⅳ（2）で③を選んだ場合に表示）
      1. 日本産科婦人科学会ARTオンライン登録データやレセプトデータに含まれない情報を示したいから
      2. 日本産科婦人科学会ARTオンライン登録データやレセプトデータより新しい情報を出せるから
      3. その他（　　　　　　　　　　　　　　　　　　　　　　　　　　）
7. **治療情報や成績の開示を行う場合、治療や登録制度に変化が生じる可能性もあります。下記の項目についてのお考えをお選びください。**

選択肢：全くそう思わない　あまりそう思わない　どちらともいえない　ある程度そう思う　非常にそう思う

- 1. 治療件数の開示について
     1. 患者の医療機関選択に有用である
     2. 情報提供の質・効率が高まる
     3. 治療の透明性が高まる
     4. 適切に治療を行っていない医療機関が淘汰される
     5. 適切に治療を行っている医療機関まで淘汰される
     6. 患者を混乱させる
     7. 患者に正しい情報が伝わらない
     8. 患者の選別が生じる（難しい症例を治療しなくなる）
     9. 治療内容が変化する
     10. 自由な治療が阻まれる
     11. 登録周期が選別されてしまう（一部の治療周期しか登録しなくなる）
  2. 多胎やOHSS等の合併症の件数や発生率の開示について
     1. 患者の医療機関選択に有用である
     2. 情報提供の質・効率が高まる
     3. 治療の透明性が高まる
     4. 適切に治療を行っていない医療機関が淘汰される
     5. 適切に治療を行っている医療機関まで淘汰される
     6. 患者を混乱させる
     7. 患者に正しい情報が伝わらない
     8. 患者の選別が生じる（難しい症例を治療しなくなる）
     9. 治療内容が変化する
     10. 自由な治療が阻まれる
     11. 登録周期が選別されてしまう（一部の治療周期しか登録しなくなる）
  3. 治療の成功率（妊娠率や生産率）の開示について
     1. 患者の医療機関選択に有用である
     2. 情報提供の質・効率が高まる
     3. 治療の透明性が高まる
     4. 適切に治療を行っていない医療機関が淘汰される
     5. 適切に治療を行っている医療機関まで淘汰される
     6. 患者を混乱させる
     7. 患者に正しい情報が伝わらない
     8. 患者の選別が生じる（難しい症例を治療しなくなる）
     9. 治療内容が変化する
     10. 自由な治療が阻まれる
     11. 登録周期が選別されてしまう（一部の治療周期しか登録しなくなる）

（1）～（3）についてその他の期待や懸念がある場合はご記入ください。

（　　　　　　　　　　　　　　　　　　　　　　　　　　　　　　　　　　　）

1. **現在の日本産科婦人科学会ARTオンライン登録データは、治療情報や成績の開示に用いるにあたってどのような問題点があると考えますか？**

選択肢：全く問題だと思わない　あまり問題だと思わない　どちらともいえない　ある程度問題だと思う　非常に問題だと思う

- - - 1. 第三者（国、都道府県、学会等）による実地監査や精度管理がないこと
      2. 電子カルテシステムとの連携など登録が効率化されていないこと
      3. 登録周期が選別される可能性があること
      4. 人工授精が含まれていないこと
      5. 登録情報だけでは症例の背景が把握できないこと
      6. 個人単位で登録されていないこと

日本産科婦人科学会ARTオンライン登録データの利用について、その他の問題点があるとお考えの場合はご記入ください。

（　　　　　　　　　　　　　　　　　　　　　　　　　　　　　　　　）

1. **適切な治療情報・成績の開示にはどのようなことが必要だと考えますか？**

選択肢：全く必要だと思わない　あまり必要だと思わない　どちらともいえない　ある程度必要だと思う　非常に必要だと思う

- - - 1. 医療機関による患者の選別が生じない仕組み
      2. ARTの適応が広がりすぎない仕組み
      3. 開示した情報を患者が誤解せず解釈するための仕組み（妊娠率を確率のみでなく、信頼区間等の範囲で示す等）
      4. 第三者機関による日本産科婦人科学会ARTオンライン登録データの管理や公開情報の管理

　　　　適切な治療情報や成績の開示にあたって、その他に必要なことがあるとお考えの

場合はご記入ください。

（　　　　　　　　　　　　　　　　　　　　　　　　　　　　　　　　　）

1. **わが国のARTの質の向上を目指す取組についてお伺いします**。**次のような方法はARTの質の向上に役立つと思いますか？**

選択肢：全くそう思わない　あまりそう思わない　どちらともいえない　ある程度そう思う　非常にそう思う

1. 個々の医療機関別の成績と全国の成績を比較した情報を公開する
2. 個々の医療機関別の成績と全国の成績を比較した情報を、個別に医療機関側に通知する
3. 日本産科婦人科学会ARTオンライン登録データやレセプトデータを用いて第三者（国、都道府県、学会等）による指導を行う

わが国のARTの質の向上を目指す取組について、その他の方法がありましたらご記入ください。

（　　　　　　　　　　　　　　　　　　　　　　　　　　　　　　　　　）

1. **不妊治療の情報提供のあり方について自由にご意見をお願いいたします。**

　　　　（　　　　　　　　　　　　　　　　　　　　　　　　　　　　　　　　　）
